# The largest Silurian vertebrate and its palaeoecological implications

**DOI:** 10.1038/srep05242

**Published:** 2014-06-12

**Authors:** Brian Choo, Min Zhu, Wenjin Zhao, Liaotao Jia, You'an Zhu

**Affiliations:** 1 Key Laboratory of Vertebrate Evolution and Human Origins of Chinese Academy of Sciences, Institute of Vertebrate Paleontology and Paleoanthropology, Chinese Academy of SciencesPO Box 643, Beijing 100044, China; 2 School of Biological Sciences, Flinders University, GPO Box 2100, Adelaide 5001, South Australia

## Abstract

An apparent absence of Silurian fishes more than half-a-metre in length has been viewed as evidence that gnathostomes were restricted in size and diversity prior to the Devonian. Here we describe the largest pre-Devonian vertebrate (*Megamastax amblyodus* gen. et sp. nov.), a predatory marine osteichthyan from the Silurian Kuanti Formation (late Ludlow, ~423 million years ago) of Yunnan, China, with an estimated length of about 1 meter. The unusual dentition of the new form suggests a durophagous diet which, combined with its large size, indicates a considerable degree of trophic specialisation among early osteichthyans. The lack of large Silurian vertebrates has recently been used as constraint in palaeoatmospheric modelling, with purported lower oxygen levels imposing a physiological size limit. Regardless of the exact causal relationship between oxygen availability and evolutionary success, this finding refutes the assumption that pre-Emsian vertebrates were restricted to small body sizes.

The Devonian Period has been considered to mark a major transition in the size and diversity of early gnathostomes (jawed vertebrates), including the earliest appearance of large vertebrate predators^1^. In contrast to the rich Devonian fossil record, gnathostomes from earlier strata have long been represented by scarce and highly fragmentary remains^2^. Traditional depictions of Silurian marine faunas typically either lack fish altogether^3^ or are dominated by diminutive jawless forms^4^. In addition to this apparent low diversity, the maximum size of pre-Devonian gnathostomes, and vertebrates in general, has been noted as being considerably smaller than later periods^1^. Until recently, the largest known Silurian gnathostomes were the osteichthyan *Guiyu*^5^ and the antiarch placoderm *Silurolepis*^6^ from the Ludlow Kuanti Formation of Yunnan, both with total body lengths of roughly 35 cm. Beyond the Silurian, the Ordovician agnathan *Sacabambaspis* from Bolivia is of comparable size^7^. The absence of pre-Devonian gnathostomes more than a few tens of centimeters in length, coupled with an apparent increase in size and diversity in the Early Devonian, has led to suggestions that jawed vertebrates were minor components of aquatic faunas prior to the Emsian^1^,^8^. Such an extended period of time with no apparent increase in body size is striking, given that the gnathostome fossil record may extend as far back as the Ordovician^9^,^10^.

Recent discoveries reveal that Silurian gnathostomes were far more diverse and widely distributed than previously recognized^10^,^11^. Of particular importance is Xiaoxiang fauna of Yunnan Province, southwestern China, based on fossils from a series of marine sediments of which the Kuanti Formation is by far the most productive^12^,^13^. This unit has produced a diverse assemblage of early fishes, including the only articulated specimens of pre-Devonian gnathostomes. Here we present a bony fish from the Kuanti Formation (Fig. 1) with an estimated length of about 1 meter, revealing that pre-Devonian gnathostomes could attain comparatively large sizes. The likely specialized predatory feeding habits of this form, and anatomical disparity to other early osteichthyans, reinforce earlier indications of a significant degree of morphological and ecological diversity among gnathostomes well before the Devonian^10^,^14^.

The apparent small size and limited diversity of Silurian gnathostomes has recently been employed as a constraint in paleoatmospheric reconstruction^1^,^8^. Models of atmospheric history based on geochemical data indicate a mid-Palaeozoic episode of global oceanic oxygenation, likely linked to the formation of a global terrestrial vascular flora and the concurrent widespread burial of organic matter^15^,^16^ and roughly coinciding with the appearance of large gnathostomes in the fossil record. Our new finding refutes suggestions that there were significant environmental constraints to vertebrate body size prior to the Emsian (~400 Ma).

## Results

### Systematic palaeontology

Gnathostomata, Gegenbaur, 1874

Osteichthyes, Huxley, 1880

Sarcopterygii, Romer, 1955

*Megamastax amblyodus* gen. et sp. nov.

### Etymology

Genus named from *megalos* and *mastax* (Greek), meaning “big mouth”. The specific epithet is derived from *amblys* and *odous* (Greek) meaning “blunt tooth”.

### Holotype

Institute of Vertebrate Paleontology and Paleoanthropology (IVPP) V18499.1, complete left mandible.

### Referred material

IVPP V18499.2, partial left mandible; IVPP V18499.3, right maxilla.

### Type locality and horizon

**Figure 1:**
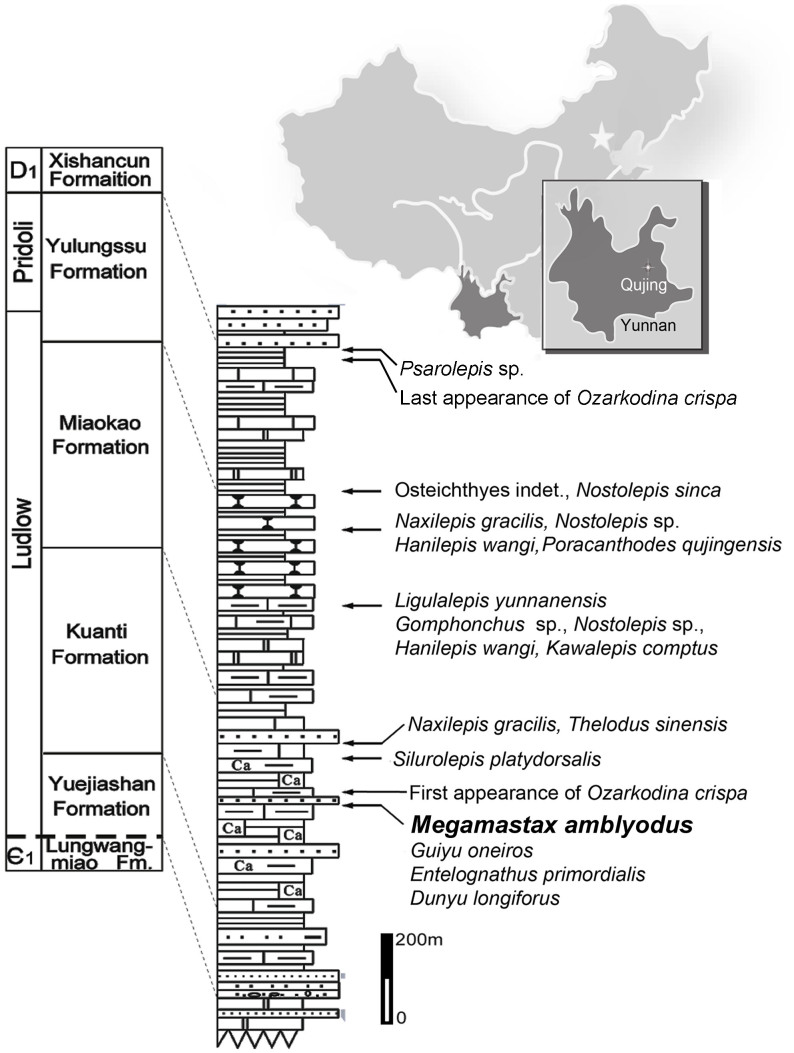
Silurian sequence in Qujing (Yunnan, China) with stratigraphic position of *Megamastax amblyodus* gen. et sp. nov. and other vertebrate taxa (modified from ref. 5, using Adobe Illustrator 10).

The Kuanti Formation, at a hill close to the Xiaoxiang Reservoir, Qujing, Yunnan, southwestern China (Fig. 1), dating to the late Ludlow (Ludfordian Stage)^11^,^12^,^13^, with a youngest age of ~423 million years ago^17^. The fossils were collected from a horizon immediately below the first appearance of the conodont *Ozarkodina crispa*. Other fishes from this horizon include the galeaspid *Dunyu*^18^, the remarkable placoderm *Entelognathus*^19^, and the osteichthyan *Guiyu*^5^,^20^.

### Diagnosis

Osteichthyan with multiple rows of closely packed conical teeth on the marginal jaw bones and widely spaced pairs of blunt teeth fused to each of the four coronoids. Coronoids fused to the lingual face of the mandible with the posterior three flanked by an elongate anterior ramus of the prearticular. Outer surfaces of the mandible and maxilla covered in cosmine with numerous embedded pores.

### Description

**Figure 2:**
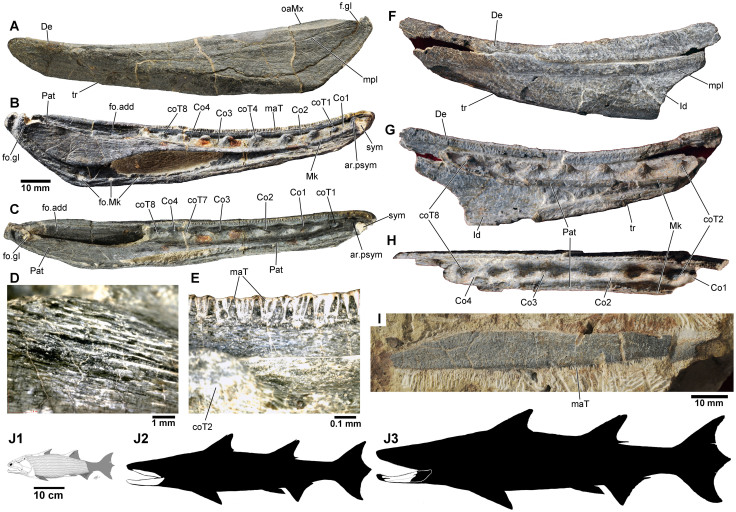
Fossils of *Megamastax amblyodus* gen. et sp. nov. (A–E) Holotype mandible (IVPP V18499.1) in (A) lateral, (B) lingular, and (C) dorsal views; close-up of prearticular bone, showing surface ridges (D), and close-up of the marginal dentition in lingual view (E). (F–H) Partial mandible (V18499.2) in (F) lateral, (G) lingular, and (H) dorsal views. (I) Right maxilla (V18499.3) in lateral view. (J) Reconstruction of (i1) *Guiyu oneiros* (ref. 13) alongside hypothetical silhouettes of (J2–3) *Megamastax* with superimposed fossil outlines (drawn by B.C.). The (J2) smaller fish is based on the V18499.1 and V18499.3, the (J3) larger on V.18499.2. ar.psym, knob-like parasymphysial structure; Co 1–4, coronoids 1–4; coT 1–8, coronoid teeth 1–8; De, dentary; fo.add, adductor fossa; fo.gl, glenoid fossa; fo.Mk, Meckelian foramen; Id, infradentary; mpl, mandibular pit line; maT, marginal teeth; oaMx, overlap area for maxilla and quadratojugal; Pat, prearticular; sym, area for parasymphysial plate; tr, indented track bordering splenial.

The external faces of the mandible (Fig. 2A, F) and maxilla (Fig. 2I) have a cosmine surface with numerous pores, as in *Achoania* and *Psarolepis*^21^. The mandible is long and low in overall shape, tapering anteriorly as in some Devonian limbed tetrapods^22^. It is gently convex in longitudinal and vertical axes, with slight medial curvature in dorsal view suggesting a narrow tapering snout. The sutured margins of the dermal bones are not clearly visible, although a small notch on the anteroventral jaw margin likely marks the posteromedial boundary of the splenial as in *Achoania* and *Psarolepis*^21^. There is a shallow semi-lunate overlap area for the maxilla and quadratojugal, while a horizontal pit-line runs almost end to end in the upper portion of the mandible. Internally, a narrow flange runs along the dorsal margin of the dentary, bearing at least two longitudinal rows of conical, slender teeth (Fig. 2E). All marginal teeth on the holotype are of roughly uniform height, but those on the inner-most row are broader and more sparsely arranged. The teeth extend almost to the tip of the jaw, well past the level of the parasymphysial articulation. On V18499.2 the marginal dentition is reduced to weathered stumps and empty tooth sockets. It is unclear if this feature is pre- or post-mortem.

**Figure 3:**
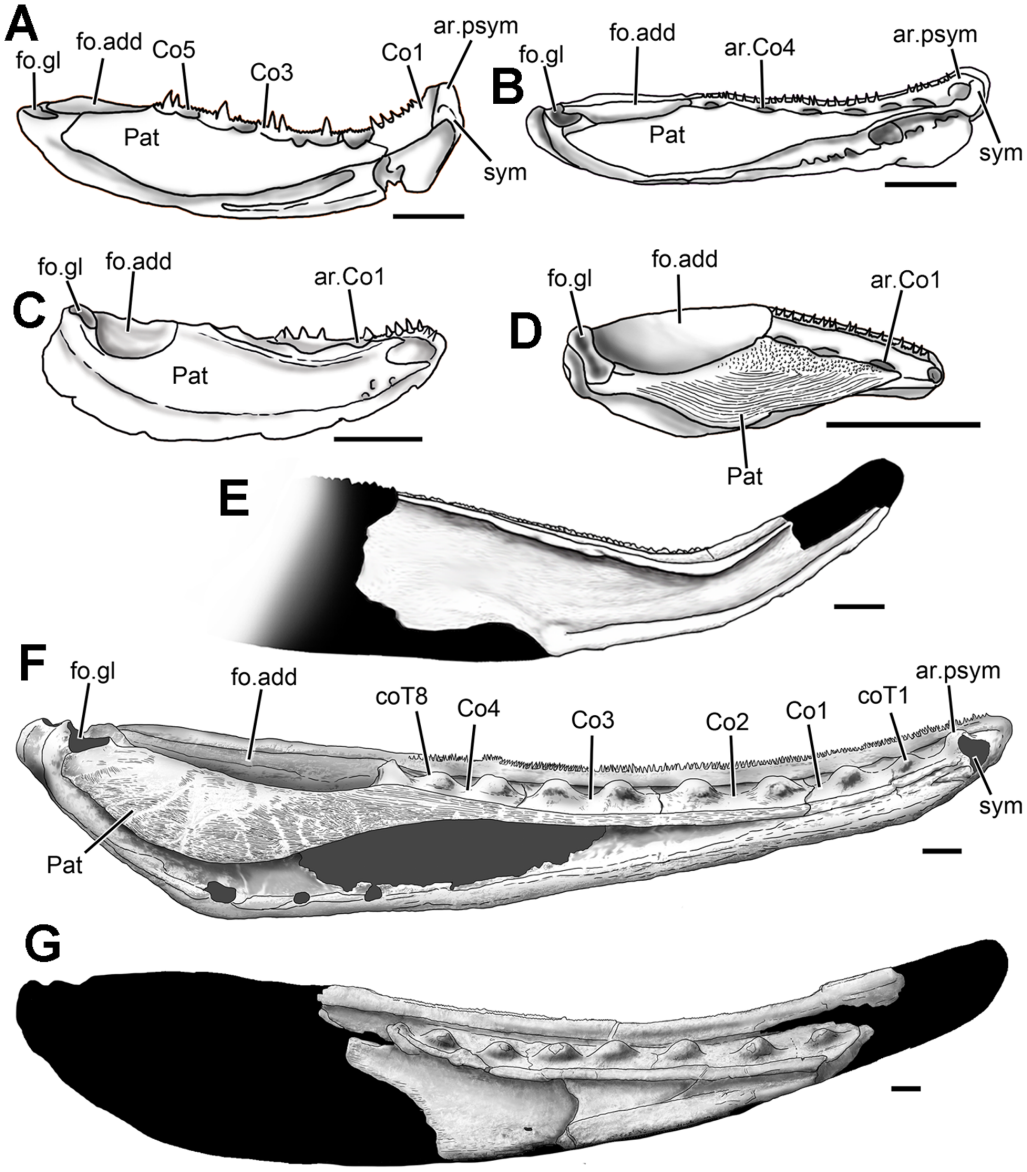
Lingual views of mandibles from selected pre-Emsian osteichthyans. Except for *Megamastax*, all are from the Lochkovian Xitun Formation, Qujing, eastern Yunnan, China. (A) *Psarolepis romeri*, IVPP V8138 (reversed). (B) *Achoania* *jarviki*, IVPP V12492.1 (reversed). (C) Jaw tentatively assigned to *Meemannia eos*, IVPP V14536.5. (D) *Styloichthys changae*, IVPP V8143.1. (E) Partial dentary of an indeterminable osteichthyan, IVPP V12493 (reversed). (F) *Megamastax amblyodus*, IVPP V18499.1 (holotype), dark grey = matrix-filled areas. (G) *Megamastax amblyodus*, IVPP V18499.2 with restored silhouette in black. (A–G) drawn by B.C. ar.psym, knob-like parasymphysial articular structure; ar.Co1–4, articulation for coronoid 1–4; Co1–5, coronoid 1–5; coT1–8, 1^st^–8^th^ coronoid tooth; fo.add, adductor fossa; fo.gl, glenoid fossa; Pat, Prearticular; sym, area for parasymphysial tooth plate. Scale bars = 5 mm.

Antero-medially there is a knob-like articular structure and symphysial overlap area for a small parasymphysial dental plate. The knob is not as strongly developed as in *Psarolepis*, *Achoania*^21^ or *Guiyu*^5^ and is concealed by the dentary in lateral view. The large prearticular is devoid of denticles, but is covered in numerous parallel ridges (Fig. 2D) as in *Styloichthys*^21^. The broad posterior section covers the dorsal and medial face of the Meckelian ossification near the adductor fossa, terminating posteriorly just behind the level of the glenoid fossa. Anteriorly, it narrows to an elongate ramus, mesially flanking the coronoid series to terminate against the posteromedial margin of the 1^st^ coronoid. The Meckelian cartilage is ossified for most of its length, although a large oval cavity anteroventral of the adductor fossa may indicate a region of incomplete ossification. The Meckelian bone extends ventrally beyond the prearticular with a series of small fenestra piercing the posteroventral margin. Posteriorly, it contributes to the rim of the adductor fossa and a small bipartite glenoid fossa. It anteriorly tapers to a narrow shelf that is fused to the knob-like parasymphysial area and the anterior tip of the prearticular. The four coronoids are smooth save for a row of widely-spaced blunt, semi-circular teeth, with two on each coronoid (Fig. 2B, C, G, H, and Fig. 3F, G). The dentition is ankylosed to a continuous median ridge, with no sockets. Tooth surfaces are smooth and lack infolding, with weathered sections on V18499.2 exposing the pulp cavity.

The 9.5 cm long maxilla (V18499.3, Fig. 2I) represents an individual of similar size to the holotype. It has identical ornamentation and corresponding contours of the occlusal margins. The biting margin is straight with no posteroventral flexion. In overall shape, the maxilla is most suggestive of porolepiforms^23^ in lacking a posterior expansion that is known in actinopterygians, onychodonts and stem sarcopterygians^5^. Multiple rows of closely packed conical teeth are arranged over the entire ventral margin.

### Comparisons

In possessing true marginal teeth, cosmine, coronoids, prearticular, and a biconcave glenoid, *Megamastax* is unambiguously an osteichthyan. The presence of cosmine, the shape of the maxilla, and the configuration of the prearticular relative to the coronoids indicate sarcopterygian affinities. Porous cosmine is found in many crown sarcopterygians^23^,^24^ as well as *Psarolepis* and *Achoania*, taxa that are usually resolved as stem sarcopterygians^25^,^26^,^27^, although a stem-osteichthyan position is also suggested^20^,^27^,^28^. While not universally distributed among sarcopterygians^24^, cosmine is unknown in actinopterygians. The maxilla lacks the pronounced posteroventral curvature and posterior expansion of *Guiyu*^5^, *Psarolepis*, onychodonts^29^,^30^ and early actinopterygians^31^,^32^,^33^,^34^, and in this respect is more similar to porolepiforms^35^. As in early sarcopterygians, the prearticular extends anteriorly to mesially flank the coronoids^5^,^21^,^36^, differing from the condition in primitive actinopterygians where the prearticular sutures against the posterior margin of the most posterior coronoid^31^,^37^.

The dentition is highly unusual. As in crown osteichthyans, the marginal teeth are discrete structures unlike the enlarged denticles of *Lophosteus* and *Andreolepis*^38^. However the marginal dentition of most early tooth-bearing osteichthyans is segregated into a single inner row of large conical teeth bordered laterally by sharpened denticles^5^,^21^,^29^,^31^,^32^,^33^,^34^. The dentary and the maxilla of *Megamastax* exhibit at least two parallel rows of sharp conical teeth of roughly uniform length. The 4-bone coronoid series of *Megamastax*, with large blunt teeth fused to the dermal surface, is unlike that of other osteichthyans where the teeth, if present, are discrete structures demarcated at the base from the adjacent bone. *Psarolepis*^21^ and *Guiyu*^5^ have five coronoids per jaw, each with sharp fangs housed in semi-lunate sockets. Those of early actinopterygians^31^, actinistians and onychodonts^29^ possess numerous minute teeth or denticles. Porolepiforms and early tetrapodomorphs have a 3-coronoid series, bearing sharp tusks with infolded surfaces and an additional row of small denticles^35^. Dipnoans lack discrete coronoids.

The coronoids of *Megamastax* share a striking similarity to the dentigerous jaw bones of some acanthodians, notably the Ischnacanthiformes and *Acanthodopsis*^39^,^40^, and to a lesser extent, the infragnathals of certain arthrodires with purported teeth^41^. As the coronoids of unambiguous stem-osteichthyans are unknown, it is unclear if this is a convergence with non-osteichthyans, or is instead a plesiomorphic relict. Examining purported ischnacanthiform jaw fragments in museum collections may yield additional early osteichthyan coronoids.

A previously described 6 cm long section of a dentary (V12493, Fig. 3E) from the Lochkovian Xitun Formation, Yunnan, is superficially similar to *Megamastax* in its large size, ornamentation and prominent marginal tooth-bearing flange^21^. It differs in the greater degree of anterodorsal curvature and in bearing only a single row of conical marginal teeth. The unpreserved coronoids were evidently not fused to the dentary. Regardless of its relationships, the specimen provides additional evidence of large osteichthyans well before the Emsian.

## Discussion

### The size of *Megamastax*

To determine the maximum size of *Megamastax* (Fig. 2J), the total length of the large but incomplete V18499.2 was extrapolated based on the complete holotype jaw, using the distance between the 2^nd^ and 8^th^ coronoid teeth as landmarks. Fusion of the dermal bones suggests that both represent adult or near-adult specimens despite the roughly 35% difference in size. V18499.2 has a preserved length of 109 mm, missing most of the posterior section, including the adductor fossa, and the front of the jaw anterior to the second coronoid tooth. The apices of the 2^nd^ and 8^th^ coronoid teeth are 70 mm apart. V18499.1 has a total mandibular length of 129 mm with a 52 mm distance between the 2^nd^–8^th^ coronoid teeth. V18499.2 is thus calculated to be 1.346 times longer than the holotype, with a restored total length of 173.65 mm (Fig. 3G).

While errors in scaling due to ontogenetic or individual variation cannot be ruled out, mandibles of *Achoania* and *Psarolepis* from the Lower Devonian Xitun Formation exhibit an even greater degree of relative size differences; the jaws of *Achoania* ranging from 32.5 to 72 mm in length^21^. While larger specimens exhibit a proportionally greater depth, due primarily to a deepening of the infradentaries, they do not exhibit consistent differences in the relative anteroposterior proportions of the glenoid fossa, adductor fossa and coronoid series, regardless of the size of the mandible^21^.

To provide an estimate for the total body length of *Megamastax*, comparisons were made with more completely known Siluro-Devonian osteichthyans (Fig. 2J). Calculations based on isolated jaws must be tentative as relative mandible-to-body size is subject to individual and ontogenetic variation. *Guiyu* is currently the only Silurian osteichthyan known from reasonably complete remains, with the holotype (V15541) measuring about 260 mm from snout-to-anal fin for a likely total length of roughly 350 mm (Fig. 2J1); the lower jaw accounting for about 1/7^th^ of that length^20^. Excluding tetrapods, Devonian osteichthyans, both sarcopterygians and actinopterygians, share a conservative fusiform anatomy with no unusually elongate or truncated body configurations. This includes *Dialipina*, an Early Devonian taxon usually resolved as a stem-osteichthyan in recent analyses^19^,^25^ and thus likely a more basal taxon than *Megamastax*, suggesting a fusiform-body via phylogenetic bracketing. Mandibular lengths in Devonian bony fishes generally account from between 1/5^th^ of body length in forms like *Strunius*^30^ and *Miguashaia*^42^ to 1/7^th^ in more elongate taxa like *Howqualepis*^34^ and *Gogosardina*^32^.

Extrapolating from this provides estimates of between 645 to 903 mm for V18499.1 with a 129 mm jaw, and between 868 to 1215 mm for V18499.2 with a 173.65 mm jaw (Fig. 2J2–3).

### The earliest durophagous predatory osteichthyan?

The coronoid teeth (Fig. 2B, C, G, H) differ from the sharp tusks of other Silurian bony fishes from the South China block, notably *Guiyu*^5^ and *Psarolepis*^21^,^27^. When coupled with the much larger size of *Megamastax*, this suggests widely divergent feeding strategies and alludes to a considerable degree of trophic specialisation well before the Devonian. Nothing is currently known of the palate, but the rounded coronoid dentition is suggestive some sort of crushing role, perhaps against a complimentary row on the dermopalatine.

**Figure 4:**
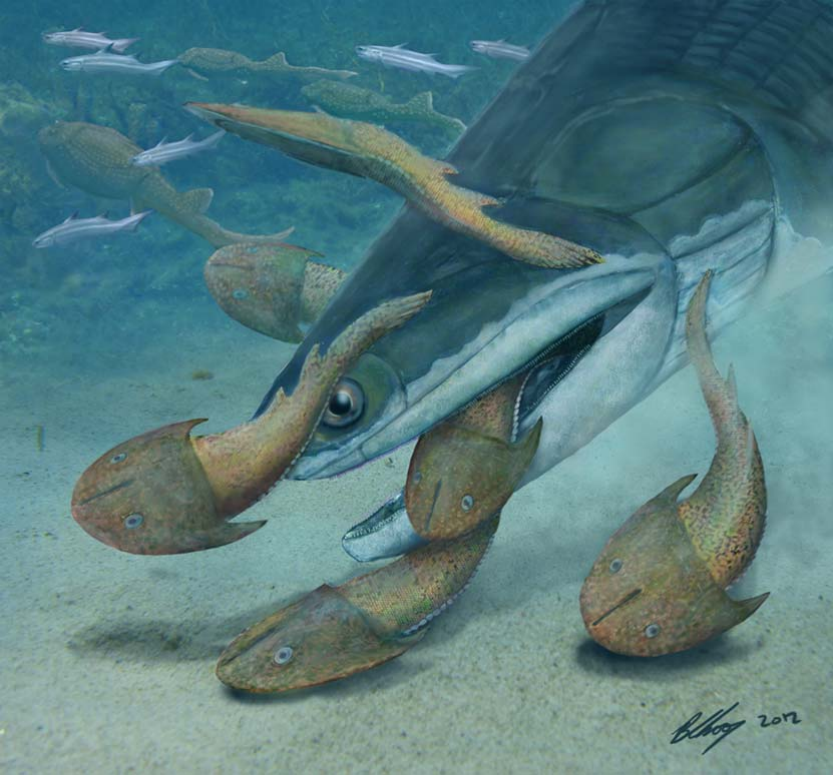
Life reconstruction of *Megamastax amblyodus* consuming the galeaspid *Dunyu longiforus* (drawn by B.C.).

Among extant fishes, dentition combining grasping and crushing morphologies is common in durophagous predators. These target hard-shelled prey, which require processing prior to injestion^43^. Such forms usually employ anterior conical teeth for initial prey capture before food is passed posteriorly to flattened or rounded molariform teeth. The shell-crushing dentition may be located on the marginal jaws as in hornsharks^44^ and wolf-eels^43^, or set within pharyngeal batteries as in many wrasses^45^. *Megamastax* differs from extant forms in that the processing dentition is on the coronoids, medial to rather than posterior to the conical teeth, which are distributed throughout the jaw margins rather than anteriorly restricted. However the contrasting tooth-form suggests a separation of activity (capture vs processing) that is broadly analogous to extant piscine durophages, possibly making it the earliest osteichthyan with specific adaptations for such a diet. The sub-tidal marine invertebrate fauna of the Ludlow of Yunnan included a rich variety of potential prey, including brachiopods, molluscs and trilobites^12^,^13^,^46^. *Megamastax* may have also consumed the heavily armoured fishes whose fossils are well represented in the Kuanti Formation (Fig. 4), including placoderms^19^ and galeaspids^18^. Given its great size, *Megamastax* could have potentially eaten any other animal in the assemblage and may thus represent the earliest vertebrate apex-predator. As an apparently specialised predator that differs substantially from contemporary osteichthyans, *Megamastax* correlates well with a documented initial increase in the functional disparity of the earliest gnathostomes which had stabilized by the Early Devonian^14^.

### Implications for palaeoatmospheric modelling

**Figure 5:**
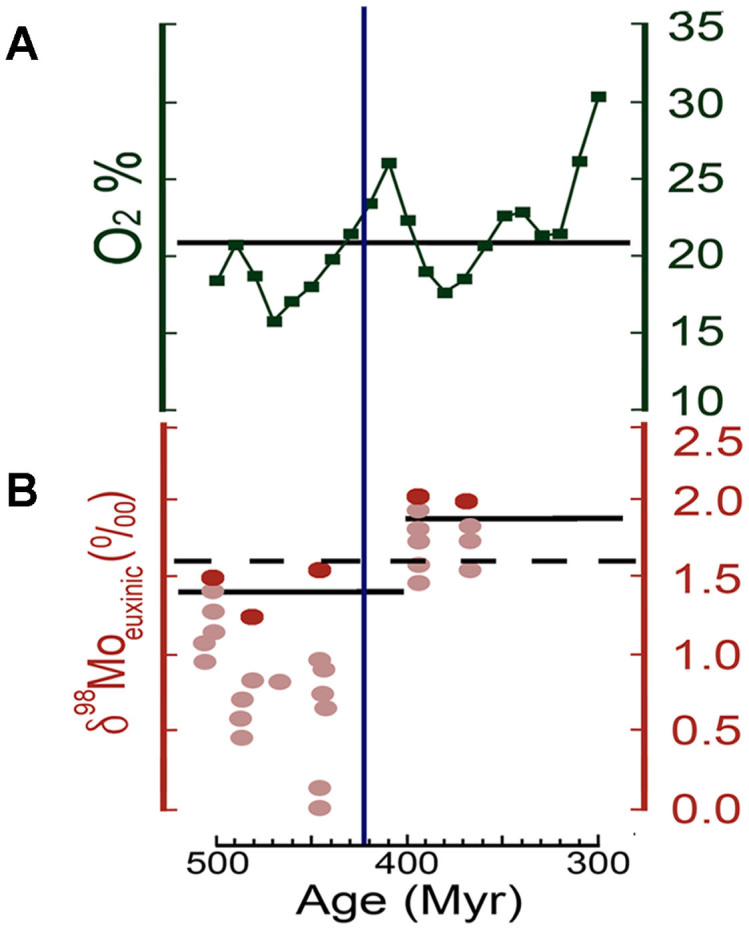
Competing models of mid-Palaeozoic oxygenation from 500 Ma to 300 Ma. Vertical blue line indicates minimum age of the Kuanti Formation and *Megamastax*. (A) From figure 2 in ref. 51. Estimates of atmospheric O2 over time based on calculations from the GEOCARBSULF model (solid line = modern O2%). (B) From figure 3B in ref. 8. Mo sediment samples with seawater (SW) values inferred from highly euxinic (red) and mildly euxinic sediments (pink). δ^98^Mo is a measure of the relative proportions of heavy and light Mo isotopes, with higher values inferring oxygenated oceans (δ^98^Mo modern SW = 2.3). Solid lines represent 90% percentiles while values above the dashed line require a substantial oxic Mo sink.

The role of oxygen availability as a significant factor in the appearance of large animals in the mid-late Palaeozoic has been the subject of considerable scrutiny, although the exact causal relationships are ambiguous and controversial due to the likely influence of other variables such as trophic tiering and cascades, temperature, and biotic interactions^47^,^48^,^49^. Recent advances in geochemistry^1^,^10^,^15^,^50^,^51^,^52^ have provided a wealth of data on early Phanerozoic climate and atmospheric conditions, allowing for correlation with key biological events. Earlier attempts at palaeoatmospheric modelling suggest consistently low Silurian O_2_ concentrations, substantially below the current atmospheric level of 21%^53^,^54^,^55^. Of the two most recent models, GEOCARBSULF^51^,^52^ is based new isotopic data of carbon and sulphur. It indicates a gradual increase of atmospheric O_2_ from the end of Ordovician with a peak exceeding modern levels towards the end of the Silurian, followed by a decrease in the Early-Middle Devonian with a low point during the Frasnian (Fig. 5A). This correlates with the relative abundance of charcoal during the Silurian to Permian^12^.

An alternative model based on molybdenum (Mo) isotopes^1^,^8^ (Fig. 5B), while with initial results spanning a broad possible time range of ~430–390 Ma, suggests a peak in the later part of the Early Devonian (~400 Ma, during the Emsian Stage) based in part on calibration with the vertebrate fossil record^1^. As body size in extant predatory marine fishes has been claimed to scale positively with both oxygen demand and uptake, with vulnerability to hypoxic mortality in large predatory forms being considerably greater than their smaller kin^1^ while fishes in general have been recorded as less tolerant of hypoxia than many marine invertebrates^56^. These observations have served as a proxy for the Emsian oxygenation scenario, with earlier limitations to oxygen availability, estimated to have been 15–50% of present atmospheric levels (PAL), imposing physiological constraints on maximal body size^8^. A date of ~400 Ma for O_2_ concentration attaining to 40% PAL (the minimum estimated requirement for predatory fishes above 1 m) was favoured when correlated against the low maximum length of Silurian gnathostomes (no taxa more than a few tens of centimeters) and the apparent rise of large predatory fishes, with presumably greater metabolic requirements, during the Devonian^1^. Although a simple causal relationship between size and hypoxia tolerance has been challenged^49^,^57^,^58^, extant marine fishes in general are also known to be less tolerant of hypoxic conditions than many marine invertebrates^1^,^56^,^59^. This suggests that low oxygen levels would have imposed some degree of extrinsic constraint on the maximum body size and available niche opportunities of the earliest gnathostomes.

Bambach^60^ proposed that the emergence of large predatory fish in the Devonian was linked to the rise of a global terrestrial flora, with an expanded trophic pyramid fuelled by phosphate-laden runoff from plant-covered continental zones. However, recent palaeobotanical discoveries have brought the timing of the evolution of vascular plants into question^61^ and indicate a well established terrestrial flora by the latest Silurian^62^. Cryptospore records suggest a floristic invasion of the land as far back as the latest Ordovician^63^. As such, the benefits of terrestrial vegetation to aquatic biotas may have been active for considerably longer than initially thought, accounting for the large size of *Megamastax* and the rich diversity in the Xiaoxiang fauna.

While it might be argued that *Megamastax*, being presumably a foraging predator of slow-moving or sessile shelled prey, likely had lower oxygen requirements than a fast midwater piscivore, it has been demonstrated that even relatively sedate benthic fishes in modern coastal communities exhibit high vulnerability to hypoxia^64^,^65^, whereas some modern foraging reef omnivores, such as the picasso triggerfish^66^, employ highly energetic forms of locomotion.

A recent time-calibrated phylogenetic analysis of a broad sample of living actinopterygians presented a striking correlation between speciation and increases in body size^67^. Based on this result, it could be argued that the large size of *Megamastax* is a simple corollary of early gnathostome diversification, rather than an indicator of extrinsic environmental factors such as oxygen level. Regardless, the existence of a metre-long predatory fish in the Ludlow raises doubts on the use of restricted vertebrate body size as a proxy for low Silurian O2 levels. This discovery does not necessarily dispute the use of Mo isotopes in palaeoatmospheric reconstruction as the ~423 Ma Kuanti Formation falls within the lower extreme of the estimated time interval of the mid-Phanerozoic peak, although it suggests that the present model requires recalibration in light of this new datum. The size of *Megamastax* and the emerging diversity of late Silurian gnathostomes based on ongoing fossil discoveries are not indicative of any significant restrictions on pre-Devonian gnathostome size and diversity. While not in itself a reliable indicator of ancient atmospheric conditions, these fossils are at least consistent with the high Silurian oxygen levels predicted by GEOCARBSULF. Given the presence of big osteichthyans in the Kuanti and Xitun formations, the purported absence of large pre-Emsian jawed fishes is seen to be a sampling artefact at least partially due to preservational and environmental biases^68^.

## Methods

All fossils are housed at the Institute of Vertebrate Paleontology and Paleoanthropology (IVPP), Chinese Academy of Sciences, Beijing. The blocks were collected from the Kuanti Formation (late Ludlow) in Qujing, Yunnan, China and prepared mechanically at IVPP using pneumatic air scribes and needles under microscopes.

### Nomenclatural acts

This published work and the nomenclatural acts it contains have been registered in ZooBank, the proposed online registration system for the International Code of Zoological Nomenclature (ICZN). The ZooBank LSIDs (Life Science Identifiers) can be resolved and the associated information viewed through any standard web browser by appending the LSID to the prefix ‘http://zoobank.org/’. The LSID for this publication is: urn:lsid:zoobank.org:pub:4FA91224-FF35-4DD1-9618-BA950DF073FE, urn:lsid:zoobank.org:act:6A4DA6A3-B675-4B4D-8F79-5CA5F9366327, and urn:lsid:zoobank.org:act:89729F30-1562-4FD9-9801-6604003C514B.

## Author Contributions

M.Z. conceived and designed the project. M.Z., W.Z., L.J., Y.Z. and B.C. did the fieldwork. B.C. and M.Z. discussed the results and wrote the main manuscript text. B.C. made illustrative drawings (figures 2J and 3) and the artistic reconstruction of *Megamastax* (figure 4). All authors reviewed the manuscript.
